# Supplementary material for: Patterns of Intron Gain and Loss in Fungi
Source: PLoS Biol. 2004 Nov 30;2(12):e422. doi: 10.1371/journal.pbio.0020422 (PMC532390; doi:10.1371/journal.pbio.0020422)
Supplement: Table S1 — Also available at http://genes.mit.edu/NielsenEtAl/. (4.3 MB ZIP). [file pbio.0020422.st001.zip › NielsenEtAl/html/799.html]

AN7500.1.NCU05225.1.MG04140.1.FG02477.1


```
 CLUSTAL W (1.82) Multiple Sequence Alignments - Introns Inserted


Sequence 1: NCU05225.1	673 aa
Sequence 2: MG04140.1	689 aa
Sequence 3: FG02477.1	693 aa
Sequence 4: AN7500.1	601 aa
Alignment Length: 710 aa
Number Identitical Residues: 347 aa
Alignment Score (without introns) 16977


MG04140.1 	MLSVGHQSRRLQGSARLITGARSQLLLNPLHTRSIASPRLHVRHALATPKITNAHARFRF
NCU05225.1	MLRT----YRVARASGLATAPRTLTLTSTTATR----------HLFTLPKSLQLRRPEKL
FG02477.1 	MPVPTMMAARLARAGQTRAINSSRCIKPSLIPRPLYSSQSRSFVKLTGASPSITKGSPVT
AN7500.1  	------------------------------------------------------------
          	                                                            

MG04140.1 	DSVH----QRQFVRYLSDVPVPRQRSRAWIIAYRAAAWFGSSIAFVGLGVVGFFLYDAST
NCU05225.1	SLIS----QRQ----LSGRPLPRTQSRLLNFGYRTAAWFGSSIAFVGLSFVAFFLYDAST
FG02477.1 	SRVTRQLPSQFFVRNLSGKPLPQGKSWILNFCYRAAAWVGISISVIGVGVVGFFIYDAST
AN7500.1  	--------------------------------------------MSSAAVIAFFIYDATT
          	                                            . . ..:.**:***:*

MG04140.1 	YKESGT-HEEIDVARTALNPRRGGPKNLPILEAYLDDNESPTARERKEKPRLVILGGGWG
NCU05225.1	YSSHATNQGDITVPKLALNPRRGGPKNLPILEIFLDDDDSEEKKKHKEKPRLVILGGGWG
FG02477.1 	YSEHPN-HSDIDVSKIALQPRSGGEKNLPIAEVYIDDDDTEEHRRLKDKPRLVILGGGWG
AN7500.1  	YRESSS-AEDIPVSELALNPRRGGPKNLPIAEVLIGDDDSEAMQAQKDKPRLVILGTGWG
          	* .  .   :* *.. **:** ** ***** *  :.*:::   :  *:******** ***

MG04140.1 	GVAILKELNPEDWNVTVISPA1NYFLFTPMLPSATVGTLELKSLVEPIRRILHRVGGHFL
NCU05225.1	SVALLKELNPDDYHVTVVSPA~NYFLFTPMLPSATVGTLELKSLVEPIRNIIDRVKGHYI
FG02477.1 	GVALLKELNPEDYHVTVISPT~NYFLFTPMLPSATVGTLELRSLVEPIRRILSRVNGHFI
AN7500.1  	SIALLKELNPGDYHVTVVSPT~NYFLFTPMLPSATVGTLGLRSLVEPVRRIVQRVHGHFL
          	.:*:****** *::***:**: ***************** *:*****:*.*: ** **::

MG04140.1 	HA1NADDVDFSHKLVEVSQKDSSGNLQRFYVPYDKLVVAVG~SSTNPHGVKGLENCFFLK
NCU05225.1	RA~AAEDVDFSSRLVEVSQKDPRGNEVRFYVPYDKLVIAVG~STTNPHGVKGLENCHFLK
FG02477.1 	RA~KAEDVDFSHKMVEVSQVDASGKDIRFYVPYDKLVIAVG~STTNPHGVKGLENAFFLK
AN7500.1  	KG~EAVDVDFSEKLVEITQINHKGEKEQFYLPYDKLVIGVG1CVTNPHGVKGLEHCHFLK
          	:.  * ***** ::**::* :  *:  :**:******:.** . **********:..***

MG04140.1 	DIRDARKIRNQIVQNLELACLPSTSDEERKRLLSFVVSGGGPTGVEFAAELFDLLNEDLT
NCU05225.1	DINDARQIRNKIIQNLELSCLPTTSDEERKRLLSFVVCGGGPTGVEFAAELFDLLNEDLT
FG02477.1 	DINDARKIRNQVIQNFELANLPTCSDEERKRLLSFCVSGGGPTGVEFAAELFDLLNEDLT
AN7500.1  	SIDDARRIKNQVLDNMELACLPTTTDEERKRLLSFVVCGGGPTGVEFASELFDLLNEDLL
          	.* ***:*:*::::*:**: **: :********** *.**********:********** 

MG04140.1 	LHFPKLLRNEISVHVIQSRGHILNTYDETVSKYAE~ERFARDQVDVLTNSRVSEVKKDRI
NCU05225.1	LHFPRLLRNEISVHLIQSRDHILNTYDEAVSKYAE~DRFSRDQVDVLVNSRVAEVRPESI
FG02477.1 	RHFPRLLRNEISVHLIQSRSHILNTYDETVSRYAE~DRFARDQVDVQTNSRVKEVLPDKI
AN7500.1  	YSFPKILRNEISVHIIQSRSHILNTYDEALSKFAE0SRFARDDVDVLTNARVKEVREDKV
          	  **::********:****.********::*::** .**:**:*** .*:** **  : :

MG04140.1 	IFTQKGPDGK-LITKELPMGFCLWSTGVS~QTEFSKKIAERLGEAQGNRHALETDSHLRL
NCU05225.1	LFTQRGPDGKTTVTKELPMGFCLWSTGVS~QAEFCKRISRQLGPAQTNRHALETDTHLRL
FG02477.1 	IFTQK-QEGGGTITKELPIGFCLWSTGVS~QTQFCQTLAAKLRKSQTNRHALETDTHLRL
AN7500.1  	VFTQL-EDGK-TVTKEIPMGFCLWSTGVG1RSDFCSRLSDKL-EAQNNKHALETDSHLRL
          	:***   :*   :***:*:*********. :::*.. :: :*  :* *:******:****

MG04140.1 	LGTPLGDVYAIGDCSTVQNNVADHIITFLQAYSWKHGKDPQTLQLHFSDWRNVAADVKKR
NCU05225.1	NGTPLGDVYAIGDCSTIQNNVADHIITFLRNLAWKHGKDPESLELHFSDWRDVAQQIKKR
FG02477.1 	NGSPMGDVYAIGDCSTVQNNVADHVVTFLRSLAWKRGKDPETLQLSFADWRGVADDVKKR
AN7500.1  	IGAPLGDVYAIGDCATVQNKVADHIVSFLRTIAWEKGKDPQKVHLTFREWRDVAARVKKR
          	 *:*:*********:*:**:****:::**:  :*::****:.:.* * :**.**  :***

MG04140.1 	FPQAASHLKRLDKLFAEFDKDQSGTLDFGELRELLRQIDSKLTSLPATAQRAHQQGQYLA
NCU05225.1	FPQATAHLKRLDKLFEEYDKDQNGTLDFGELRELLKQIDSKLTSLPATAQRAHQQGQYLA
FG02477.1 	FPQAVGHLKRVDKLFEEFDKDKSGTLDFDELTQLLRNVDNKLTSLPATAQRAHQQGQYLA
AN7500.1  	FPQASNHLRRLDRLFEQYDKDHSGTLDFEELSELLHQIDTKLTSLPATAQRANQQGQYLG
          	****  **:*:*:** ::***:.***** ** :**:::*.************:******.

MG04140.1 	HKLNKLARAAPGLRANDIMDGDVDAAVYKAFEYKHLGSLAYIGNSAVFDWGEGWSLTGGL
NCU05225.1	HKFNKLARAAPGLSANEIHEGDLDAAVYKAFEYRHLGSLAYIGNSAVFDWGDGWNFSGGL
FG02477.1 	RKFNRMTRLHEGLDANDIRDGDVDAAVYKAFEYKHLGSLAYVGNSAIFDWGEGRSIAGGL
AN7500.1  	RKLTKIAAAMPGMQANQIDYGDLDEAVYKAFNYKHLGSLAYISNAAIFDFG-GLNFGGGV
          	:*:.:::    *: **:*  **:* ******:*:*******:.*:*:**:* * .: **:

MG04140.1 	WAVYAWRSVYFAQSVSFRTRMLLAMDWLKRGLFGRD1LMAY---------------
NCU05225.1	WAVYAWRSIYFTQSVSLRTRVLMAMDWGKRALFGRD1LMSY---------------
FG02477.1 	WAVYAWRSVYFAQSVSLRTRLLMAMDWTKRGLFGRD1LMSF---------------
AN7500.1  	LAMYLWRSVYFAESVSLRTRVMLAMDWAKRALFGRV1YCSMGMQSAISHRLCQSSY
          	 *:* ***:**::***:***:::**** **.****    : . .:: :   ..::
```
